# Nutrition in Pediatric Inflammatory Bowel Disease: From Etiology to Treatment. A Systematic Review

**DOI:** 10.3390/nu8060334

**Published:** 2016-06-01

**Authors:** Francesca Penagini, Dario Dilillo, Barbara Borsani, Lucia Cococcioni, Erica Galli, Giorgio Bedogni, Giovanna Zuin, Gian Vincenzo Zuccotti

**Affiliations:** 1 Pediatric Department, “V. Buzzi” Children’s Hospital, University of Milan, Via Castelvetro 32, 20154 Milan, Italy; dario.dilillo@icp.mi.it (D.D.); barbara.borsani1@gmail.com (B.B.); lucia.cococcioni@gmail.com (L.C.); egsorridi@gmail.com (E.G.); giovanna.zuin@icp.mi.it (G.Z.); gianvincenzo.zuccotti@unimi.it (G.V.Z.); 2 Clinical Epidemiology Unit, Liver Research Center, Basovizza, 34012 Trieste, Italy; giorgiobedogni@gmail.com

**Keywords:** nutrition, inflammatory bowel disease, children, etiology, treatment

## Abstract

Nutrition is involved in several aspects of pediatric inflammatory bowel disease (IBD), ranging from disease etiology to induction and maintenance of disease. With regards to etiology, there are pediatric data, mainly from case-control studies, which suggest that some dietary habits (for example consumption of animal protein, fatty foods, high sugar intake) may predispose patients to IBD onset. As for disease treatment, exclusive enteral nutrition (EEN) is an extensively studied, well established, and valid approach to the remission of pediatric Crohn’s disease (CD). Apart from EEN, several new nutritional approaches are emerging and have proved to be successful (specific carbohydrate diet and CD exclusion diet) but the available evidence is not strong enough to recommend this kind of intervention in clinical practice and new large experimental controlled studies are needed, especially in the pediatric population. Moreover, efforts are being made to identify foods with anti-inflammatory properties such as curcumin and long-chain polyunsaturated fatty acids *n*-3, which can possibly be effective in maintenance of disease. The present systematic review aims at reviewing the scientific literature on all aspects of nutrition in pediatric IBD, including the most recent advances on nutritional therapy.

## 1. Introduction

Nutrition is involved in several aspects of pediatric inflammatory bowel disease (IBD), ranging from disease etiology to induction and maintenance of disease. The etiology of inflammatory bowel disease (IBD) is believed to be multifactorial, caused by an interplay of genetic, environmental, microbial, and immunological factors. Among the environmental risk factors, dietary elements have received considerable attention in recent years. Epidemiological studies have demonstrated a rising incidence of IBD in countries where the disease is more prevalent, such as Europe and North America, as well as in areas where IBD was previously thought to be uncommon [1]. This increase has occurred with the spread of the “Western” diet, which is high in fat and protein but low in fruit and vegetables [2,3]. Numerous studies, conducted mainly in adults, assessed whether there are associations between the development of IBD and diet and suggested that specific nutrients may play a role as risk factors or protective factors for the development of disease [3,4,5,6,7,8]. However, it must be noted that many of these studies rely on food frequency questionnaires, which have a poor accuracy due to recall bias. The hypothesis of dietary factors influencing gut inflammation may be explained through several biological mechanisms, including antigen presentation, change in prostaglandin balance, and alteration of the microflora [2,9]. Aside from etiology, nutrition plays a significant role in treatment of disease. Among the traditional nutritional approaches, exclusive enteral nutrition (EEN) is an extensively studied, well established, and valid approach to remission of pediatric Crohn’s disease (CD). The latter nutritional therapy presents several advantages including control of inflammatory changes, mucosal healing, positive benefits to growth and overall nutritional status, and avoidance of other medical therapies. Aside from EEN, recently several new nutritional approaches are emerging and have seemed to be successful. The present systematic review aims at reviewing the scientific literature on all aspects of nutrition in pediatric IBD, including the most recent advances in nutritional therapy.

## 2. Methods

**Figure 1:**
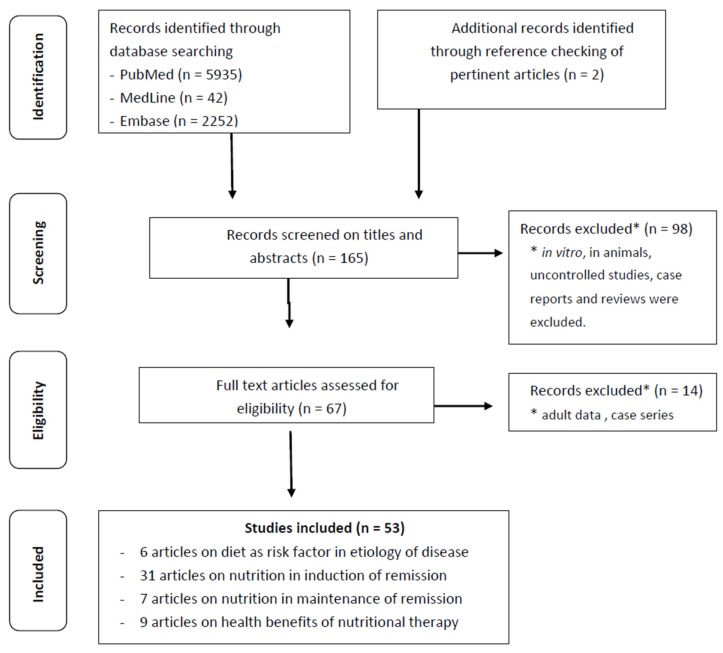
Flow-chart on the methods of the systematic review.

A structured literature search was performed by two investigators (Francesca Penagini and Barbara Borsani) independently, in PubMed, EMBASE, and Medline starting from January 1982 up to April 2016 using the following keywords: “Pediatric Inflammatory Bowel Disease”, “Pediatric IBD”, “Inflammatory Bowel Disease in children”, “IBD in children” in any combination with “diet”, “dietary intakes”, “nutrition” and cross referenced with “etiology”, “induction of remission”, “relapse” and “treatment”. The search was limited to full-text papers in English and resulted in a total of 8229 articles. By screening titles and abstracts, studies performed *in vitro*, in animals, uncontrolled studies, case reports, and reviews were excluded. Duplicate studies were removed. Eligible papers were cross-checked for references, which resulted in two additional papers. Figure 1 shows the flow-chart of our systematic review. For randomized clinical trials the absolute risk change with exact 95% confidence intervals (CI) was calculated. For cohort studies, the incidence rates with exact 95% CI were calculated.

## 3. Nutrition in Etiology of Pediatric Inflammatory Bowel Disease

Data on diet as a risk factor for the onset of pediatric IBD are scarce. There are few studies in the literature apart from case-control studies, which have shown a potential role of some dietary elements as risk factors for disease onset in pediatric IBD. Nevertheless, these studies present methodological limitations such as the use of self-administered patient questionnaires, therefore recall bias. Table 1 summarizes the main studies on nutrition factors in etiology of pediatric IBD.

The Japanese Epidemiology Group of the Research Committee of IBD conducted a multisite, hospital-based, case-control study to examine the environmental risk factors for UC in 101 patients who were 10–39 years old at the time of disease onset [10]. Information was obtained from self-administered patient questionnaires. Combined consumption of Western foods (bread for breakfast, butter, margarine, cheese, meats, and ham and sausage) was significantly related to an increased risk of UC Relative risk (RR) for low consumption of Western foods 1.0 (CI not available), for intermediate consumption RR = 1.9; 95% CI 1.0 to 3.7, for high consumption RR = 2.1; 95% CI 1.0 to 4.1, *p* = 0.04. Margarine (as an individual Western food item) was positively associated with UC (RR for low consumption: 1.0 (CI not available), for intermediate consumption RR = 1.2; 95% CI 0.6 to 2.4, for high consumption RR = 2.6; 95% CI 1.4 to 5.2; trend, *p* = 0.005. There was also a tendency towards positive association of bread for breakfast with UC. For low consumption RR = 1.0, for intermediate consumption RR = 0.7; 95% CI 0.4 to 1.4, for high consumption RR = 2.1; 95% CI 1.0 to 4.3, *p* = 0.07. The risk did not measurably vary with the consumption of typical Japanese foods, vegetables and fruits, confectioneries, or soft drinks (RR data not available) [10].

The group of Amre *et al.* [11] conducted a case-control study evaluating the pre-illness diet of new onset CD in Canadian children (age < 20 years, *n* = 103) using a validated food-frequency questionnaire (FFQ) in comparison to healthy controls matched for age and gender (*n* = 202). The FFQ was administered to the cases within one month of diagnosis and investigated dietary habits within the 12 months prior to disease diagnosis. Results showed negative associations with vegetables (OR = 0.69; 95% CI 0.33 to1.44, *p* = 0.03), fruits (OR = 0.49; 95% CI 0.25 to 0.96, *p* = 0.02), dietary fiber (OR = 0.12; 95%, CI 0.04 to 0.37, *p* < 0.001), and fish (OR 0,46, 95% CI 0.20–1.06, *p* = 0.02); consumption of long-chain ω-3 fatty acids was negatively associated with CD (OR = 0.44; 95%, CI 0.19 to 1.00, *p* < 0.001), whereas positive associations were evident for total fats (OR = 2.30; 95% CI 0.67 to 7.96, *p* = 0.15), monounsaturated (OR = 2.41; 95% CI 0.72 to 8.07, *p* = 0.08), and saturated (OR = 1.81; 95% CI 0.59 to 5.61, *p* = 0.40) fats, even though these associations were not statistically significant. The authors concluded that children who consumed higher amounts of fruits were at lower risk for CD (*p* = 0.02), as well as children who consumed higher amounts of fish and nuts, rich in long-chain ω-3 fatty acids such as docosahexaenoic acid (DHA), eicosapentaenoic acid (EPA) and docosapentaenoic acid (DPA) (OR = 0.44; 95% CI 0.19 to 1.0, *p* = 0.03). When the ratio of long chain ω-3/arachidonic acid was evaluated, a higher ratio was associated with a significantly reduced risk for CD (OR = 0.32; 95% CI 0.14 to 0.71, *p* = 0.02) [11]. In a similar case-control study, D’Souza *et al.* [12] investigated the role of specific dietary patterns in the risk for developing pediatric CD (*n* = 149, mean age at diagnosis 13.3 ± 2.6 years). They observed that dietary patterns characterized by meats, fatty foods, and desserts were positively associated with pediatric CD in both genders (OR = 4.7, 95% CI 1.6–14.2); on the contrary, dietary patterns characterized by vegetables, fruits, olive oil, fish, grains, and nuts were inversely associated with CD in both genders (girls OR = 0.3; 95% CI 0.1 to 0.9; boys OR = 0.2; 95% CI 0.1 to 0.5).

More recently, Jacobsen *et al.* [13] have investigated environmental risk factors for onset of IBD in children. The authors included 118 IBD patients aged < 15 years and 447 healthy controls. Risk factors were investigated by means of a questionnaire. Results showed that a high sugar intake was a risk factor for IBD (IBD OR = 2.5; 95% CI 1.0 to 6.2, CD OR = 2.9; 95% CI 1.0 to 8.5); while protective factors were daily vegetable consumption (CD OR = 0.3; 95% CI 0.1 to 1.0), UC OR = 0.3; 95% CI 0.1 to 0.8) and wholemeal bread consumption (IBD OR = 0.5; 95% CI 0.9 to 0.9), CD OR = 0.4; 95% 0.2 to 0.9) [13].

There are plausible biological mechanisms that can explain the abovementioned role of dietary factors in IBD pathogenesis. The *n*-3 polyunsaturated fatty acids (PUFAs) are present mainly in fish and can be synthesized in humans from alpha-linolenic acid, an essential fatty acid. EPA is a constituent of cell membranes and is metabolized to prostaglandin E3 and leukotriene B5, both of which are less pro-inflammatory than eicosanoids derived from *n*-6 PUFAs. A diet high in EPA may therefore protect the colonic mucosa against inflammation [7]. Plausible mechanisms exist to support the association between fiber intake and risk of CD. Anaerobic bacterial fermentation of undigested dietary carbohydrates and fiber polysaccharides produces short-chain fatty acids (SCAFs) like acetate, propionate, and butyrate, which are involved in the maintenance of colonic homeostasis. These molecules are utilized as fuel sources for colonocyte metabolism and seem to have an anti-inflammatory effect on epithelial cells, through inhibition of the production and release of inflammatory mediators. Consequently, a diet poor in fiber and lower in SCFAs may cause a disruption of the homeostasis between bacteria and colonocytes, leading to intestinal inflammation [16].

## 4. Nutrition in Induction of Remission in Pediatric Inflammatory Bowel Disease

The first reports on the successful use of enteral nutrition in induction of remission in pediatric CD date back to the 1980s [17,18]. Since then, exclusive enteral nutrition (EEN) with specific formulas has been thoroughly studied and has become a well-established nutritional approach shown to alleviate clinical symptoms, induce mucosal healing, improve nutritional status, and normalize laboratory parameters associated with active inflammation in pediatric CD. These data have been confirmed in two meta-analyses demonstrating that EEN is as effective as corticosteroids in inducing disease remission in pediatric CD [19,20], achieving normalization of inflammatory markers and clinical remission rates in >80% of subjects [21] irrespective of disease phenotype [22].

Studies on the efficacy of EEN in active CD are reported in Table 2, a comparison between two different nutritional regimens is given in Table 3, and studies comparing the efficacy of EEN on corticosteroids in active CD are reported in Table 4. As the included studies were significantly heterogeneous for study design, outcomes observed, and sample size, a meta-analysis was not performed.

More recently, novel nutritional approaches have been proposed and their efficacy has been proved in case series and dietary intervention studies [48,49,50,51]. Table 5 summarizes the main studies on the novel nutritional approaches for induction of remission in pediatric IBD. Among the novel approaches, the specific carbohydrate diet (SCD) has gained attention. It restricts complex carbohydrates and eliminates refined sugar from the diet, based on the rationale that the sugars and complex carbohydrates are malabsorbed and could cause alterations in microbiome composition, contributing to the intestinal inflammation of IBD. The clinical efficacy in inducing remission of symptoms and improvement of laboratory parameters has been demonstrated by reviewing medical records and in a prospective trial. Suskind *et al.* [49] reviewed medical records of children and adolescents with CD (*n* = 10) aged 7–16 years, treated with the SCD for a period of 5–30 months. Symptoms of all patients were resolved at a routine clinic visit three months after initiating the diet. Laboratory indices and fecal calprotectin either normalized or significantly improved at the follow-up clinic visits. It must be noted that the study was retrospective, hence the observed events (improvement of symptoms, laboratory parameters, and fecal calprotectin) could be due to chance. Cohen *et al.* conducted a trial evaluating clinical improvement and mucosal healing in children and adolescents with active CD, (mean age 13.6 years (*n* = 9)) after a 12–52 week trial of a SCD. The study showed at both the 12-week endpoint and the 52-week extension period clinical improvement assessed by PCDAI. In 6/10 patients (60%) remission was achieved by week 12. Mucosal healing (Lewis score < 135) was observed in 40% (4/10) of patients at week 12, 80% of patients showed significant mucosal improvement at week 12 when compared to baseline (*p* = 0.012) [50]. Aside from SCD, another novel dietary intervention has been proposed and studied in a prospective randomized trial by Sigall-Boneh *et al.* [51]. The new approach consists of 50% caloric intake with an exclusion diet specific for CD and 50% intake with a polymeric formula for a period of six weeks. The authors studied children and young adults (*n* = 47, 34 children) with active CD and with a mean age of 16.1 ± 5.6 years. The specific CD diet excludes many types of foods, including dairy products, margarine, gluten-containing cereals, smoked products (meat and fish), maize and potato flour, sauces, snacks, fruit juices, chocolate, coffee, and alcohol. Response and remission was obtained in 37 (78.7%) and 33 (70.2%), patients, respectively. Remission was obtained in 70% of children and 69% of adults.

The effect of a cow’s milk protein (CMP) elimination diet on the induction and maintenance of remission in children with ulcerative colitis (UC) has been assessed in a randomized controlled trial by Strisciuglio *et al.* [52]. The authors randomized children to receive a CMP elimination diet or a free diet associated with concomitant steroid induction and mesalazine maintenance treatment. No significant differences were noted between the two groups in frequency of induction of remission and frequency of relapse.

## 5. Nutrition in Maintenance of Remission in Pediatric IBD

Exclusive Enteral Nutrition (EEN) is recommended as first line therapy to induce remission in pediatric CD [53], but only few studies analyzed the effect of dietary intervention for IBD maintenance of remission. New approaches recently proposed consider the elimination of foods thought to be noxious, through exclusion diets, or the supplementation of anti-inflammatory substances. Some data show that maintenance enteral nutrition (MEN) could reduce relapse rates. Table 6 summarizes the main studies focusing on enteral nutrition in maintenance of disease remission in pediatric IBD. In a study of Belli *et al.* [54], a small group of children continued intermittent EEN via nasogastric tube (NT) for one month out of four over the course of one year, showing improvement in CD clinical activity and growth [2]. Recently, in a retrospective study, Duncan *et al.* [55] analyzed 59 children with newly diagnosed CD, showing that the remission rate at one year was significantly higher in patients continuing MEN 60% (9/15), compared to 15% (2/13) in patients taking no treatment (*p* = 0.001) and with patients taking azathioprine (*p* = 0.14). Referring to Partial Enteral Nutrition (PEN), data about maintenance of remission in pediatric IBD are scarce. Evidences from adult population suggest that this kind of treatment could reduce the risk of relapse and improve the response to biological drugs [55,56,57,58,59,60]. A report of Wilschanski *et al.* [61] studied the effect of providing supplementary enteral nutrition, in addition to normal diet, in children and adolescents affected by CD after induction of remission with EEN. The study demonstrated fewer relapses and improvements of linear growth in children who continued supplementary feeding respect to control group [61]. Considering new nutritional interventions, a lacto-ovo vegetarian diet, also called a semi-vegetarian diet (SVD), has been evaluated in a prospective study on patients affected by CD, showing a significant effect on relapse prevention compared to an omnivorous diet (remission rate at two years 94% (15/16) for SVD *vs.* 33% (2/6), *p* = 0.0003) [62]. Another kind of exclusion diet has been attempted by Obih *et al.* [63] in a retrospective study including pediatric patients affected by CD or UC. A group of children followed the Specific Carbohydrate Diet (SCD) for a period between three and 48 months (mean of 9.6 ± 0.1 months); some of the patients were treated in conjunction with medical therapy, while the second group was treated only with standard medical therapy. The SCD group showed improvement of clinical and inflammatory markers, but the compliance to SCD was often poor and some of the patients who followed the SCD experienced weight loss [63].

Beyond nutritional therapy for maintenance of remission through exclusion diets, efforts are being made to evaluate the possible efficacy of supplementation of anti-inflammatory substances. One of the most studied is diferuloymethane, also known as curcumin. It is the most important component of the plant *Curcuma longa* or turmeric, belonging to the family Zingiberaceae. This kind of plant is typical of India, China, and Sri Lanka [64]. It is used in Indian and Chinese traditional medicine and it appears to have many properties such as anti-inflammatory, antioxidant, anti-tumor, antiplatelet, antibacterial, and antifungal effects [65,66]. Pathogenic mechanisms that explain all the curcumin properties are complex and partially known, but evidences suggest that the modulation of the NF-kB pathway has a pivotal role [67].The therapeutic use of curcumin is limited by its unfavorable pharmacokinetics, but oral administration of the drug reaches active levels in the gastrointestinal tract, so this natural substance could be useful for treating gut diseases [68]. Recent studies demonstrate that curcumin may be used as an adjunctive therapy for maintenance of remission in IBD, in particular in UC. Hanai *et al.* [69] evaluated the efficacy of curcumin as additional therapy for maintenance of remission in patients between 13 and 65 years with quiescent UC and consuming mesalazine or sulfasalazine. In this randomized, multicenter, double-blind, placebo-controlled trial, a group of patients receiving 2 g curcumin per day for six months has been compared with a group treated with a placebo. During the study period, relapses were lower in patients taking curcumin than in the placebo group (2/43 (4.65%) in curcumin group *vs.* 8/39 (20.51%) in placebo group, *p* = 0.049). Moreover, the authors noted improvements in the Clinical Activity Index (CAI) and the Endoscopic Index (EI) (secondary pre-specified outcomes) in patients receiving curcumin (CAI, *p* = 0.038 and EI, *p* = 0.0001) [69]. Finally, this natural anti-inflammatory agent seems to have high tolerability, without severe adverse effects in the pediatric population. The average intake of curcumin in countries where it is traditionally used in the diet, such as in India, is approximately 60–100 mg of curcumin daily in an adult individual [70]. Suskind *et al.* [71] enrolled 11 patients between 11 and 18 years affected by CD or UC in a tolerability study. During the trial, the curcumin dose was gradually increased from 500 mg twice a day to 2 g twice a day and continued for three weeks, without significant side effects [71].

Great interest has also been shown in *n*-3 PUFAs and their pleiotropic effects. The longer chain *n*-3 PUFAs, represented by EPA, DHA, and DPA, are mainly contained in fish oil and have numerous properties such as anti-inflammatory, anti-thrombotic, anti-arrhythmic, hypolipidemic, and vasodilatory activity [72,73]. Their anti-inflammatory effect, especially, seems to be due to *n*-3’s role in the arachidonic acid pathway and the subsequent downregulation of proinflammatory cytokine synthesis, decreased leukocyte chemotaxis, and decreased T cell reactivity [74,75]. During the last decades, the idea that an *n*-3 rich diet could have positive effects in patients affected by IBD has been evaluated in numerous studies, particularly in the adult population, with conflicting results [76,77,78]. Meister *et al.* have studied *in vitro* the influence of a fish oil enriched enteral diet on intestinal tissues taken from CD, UC, and non-IBD control patients, showing that the anti-inflammatory effect of fish oil is significantly more marked in UC compared with CD [79]. Some evidence has shown clinical improvement after supplementation of *n*-3; however the most robust evidence is from two randomized double-blind, placebo-controlled studies on large adult populations affected by CD; these have not demonstrated any significant effects of *n*-3 supplementation in relapse prevention [80], and a recent Cochrane review has concluded that omega 3 fatty acids are probably ineffective for maintenance of remission in CD [81]. Concerning pediatric IBD, an Italian double-blind, randomized, placebo-controlled study showed a significant effect on reduction of relapse. The number of patients who relapsed at 12 months was significantly lower in the Omega-3 fatty acid group (group 1) compared to patients receiving a placebo (group 2) (relapse rate group 1 11/18 (61%), group 2 19/20 (95%); *p* < 0.001 [82]. In conclusion, although Omega-3 appears to be safe, except for mild gastrointestinal tract symptoms (diarrhea, unpleasant taste, bad breath, heartburn, and nausea), further studies are needed to confirm the real efficacy of *n*-3 for IBD treatment in pediatric population. Table 4 summarizes the main pediatric studies on nutrition in maintenance of disease remission.

## 6. Health Benefits of Nutritional Therapy in Pediatric IBD

With respect to adult forms, pediatric IBD have specific features, not only for severity and extension, but also because the disease involves growing individuals, with possible irreversible consequences in adult life. Weight loss and failure to thrive, delayed puberty, and low bone mineral density are frequent signs in children affected by IBD, in particular in CD, because of malabsorption and malnutrition. The etiology of poor nutritional status in children affected by IBD is complex and involves reduced dietary intake, increased gastrointestinal nutrient losses, increased energy requests due to inflammation, and altered metabolism [83,84]. Moreover, the linear growth and nutritional status of children affected by IBD are also compromised by immunosuppressant drugs, especially corticosteroids, used for the treatment of the disease [85]. All these reasons justify the particular importance of nutritional therapy in pediatric IBD. EEN is at least as efficacious as corticosteroids in inducing remission in pediatric CD, but it has minimal side effects and several advantages in terms of mucosal healing, restoration of nutritional status, bone health, and liner growth in children [53]. Multiple studies demonstrate that, with respect to conventional immunosuppressant drugs, nutritional therapy improves weight gain, height velocity, body composition, and insulin-like growth factor (IGF)-1 and IGF binding protein 3 (IGFBP-3) [18,54,61,86,87,88,89,90]. Table 5 illustrates the main studies on the health benefits of nutritional therapy in pediatric IBD. Berni Canani *et al.* [44], in a retrospective study on 47 children with active CD, compared the effect of EEN *versus* corticosteroid therapy. Children treated with EEN for eight weeks showed higher serum iron and albumin levels, and more pronounced linear growth than group treated with corticosteroids [44]. Moreover, bone health seems to benefit from nutritional therapy too. The pathogenesis of bone loss in pediatric IBD is complex: malnutrition, compromised linear growth, lean mass deficit, delayed puberty, menstrual irregularity, reduced physical activity, persistent inflammation, and prolonged use of corticoids are the major factors responsible [91,92,93]. Although the direct effect of EEN in increasing bone mineral density (BMD) has been poorly studied, Whitten *et al.* demonstrated that the normalization of bone turnover markers in children was affected by CD after eight weeks of treatment with EEN [6]. In addition, in the latest clinical guidelines about skeletal health issued by ESPGHAN and NASPGHAN, the authors affirm the benefits of enteral nutrition. In terms of linear growth and lean mass, EEN or supplemental enteral nutrition could induce improvement of bone health, and they recommend these tactics not only during the acute phase, but also during remission phases of IBD, particularly in children and adolescents with delayed linear growth [7]. Table 7 shows the main studies on the health benefits of nutritional therapy in pediatric IBD.

## 7. Strengths and Limitations

The main strength of the present systematic review is the rigorous methodological assessment and analysis of the literature using pre-specified criteria. The methods we used helped to reduce the risk of bias (thorough literature research performed by two researchers independently and duplicate data abstraction). Nevertheless, some limitations should be pointed out. In the first instance, the methodological quality of included trials was not high and some trials had small sample sizes. Moreover, a significant limitation lies in the lack of standardization of outcome measures, clinical heterogeneity of subjects enrolled, variation in the length of follow-up and in the duration of interventions, and the use of concomitant medications.

## 8. Conclusions

Nutrition has a significant role in pediatric IBD. With regards to etiology, the available pediatric data suggest that dietary patterns characterized by consumption of meats, fatty foods, desserts, and a high sugar intake are associated with an increased risk of disease. Likely protective factors are represented by consumption of vegetables, fruits, fish, olive oil, and wholemeal bread. Large scale randomized controlled clinical trials are needed to confirm these data.

For treatment of disease, EEN is the only nutritional approach with robust evidence of effectiveness in induction of remission; PEN is known to be advantageous in reducing relapse rates. Besides the known elemental, semi-elemental, and polymeric formulas, the scientific community is currently looking into modifying dietary habits to maintain disease remission mainly through exclusion diets: the Specific Carbohydrate Diet and the Crohn’s Disease exclusion diet are now in the spotlight. Nevertheless, the poor compliance to nutritional intervention and the risk of nutritional deficits when exclusion diets are too strict are factors that have to be considered when these kinds of treatments are proposed. Despite the promising results of the novel nutritional approaches, the available data are not strong enough to recommend these interventions in clinical practice and new large controlled studies are needed, especially in pediatric population, to confirm these data.

## Figures and Tables

**Table 1:** Dietary factors and etiology in pediatric IBD. IBD = inflammatory bowel disease, UC = ulcerative colitis, CD = Crohn’s disease, LC = long chain.

Dietary Factors and Etiology in Pediatric IBD			
Author/Year	Study Type	Population	Main Findings
Gilat *et al.* 1987 [14]	Case-control study	Patients with IBD (*n* = 499; UC = 197, CD = 302) aged < 25 years with disease onset before 20 years of age. For each patient two age and sex matched health controls.	- Patients with CD and UC consumed significantly lower fruits and vegetables than controls (*p* < 0.01). For UC: low consumption (0 and <1/day) *vs.* high consumption (1–3 and >4/day) OR = 0.77; 95% CI 0.45 to 1.35. For CD: low consumption (0 and <1/day) *vs.* high consumption (1–3 and >4/day) OR = 0.58; 95% CI 0.37 to 0.91. - No significant differences were found between patients and controls in the frequency of breast feeding (*p* < 0.01), cereal consumption (*p* < 0.01) and sugar added to milk in infancy (*p* < 0.01).
Japanese Epidemiology Group of the Research Committee of IBD, 1994 [10]	Case-control study	Patients with UC (*n* = 101) who were aged 10–39 years at the time of disease onset. Healthy control subjects (*n* = 143).	- Combined consumption of Western foods (bread for breakfast, butter, margarine, cheese, meats, and ham and sausage) was significantly related to an increased risk of UC (Relative risk (RR) for low consumption 1.0, RR for intermediate consumption 1.9; 95% CI 1.0 to 3.7, RR for high consumption 2.1, 95% CI 1.0 to 4.1; trend, *p* = 0.04). - Margarine (as an individual Western food item) was positively associated with UC (trend, *p* = 0.005).
Baron *et al.* 2005 [15]	Case-control study	IBD patients (*n* = 282; CD 222, UC 60) with onset before 17 years of age and healthy controls matched for age, sex, and geographical location (*n* = 282).	- Breastfeeding either partially or exclusively was a risk factor for CD (CD OR = 2.1; 95% CI 1.3 to 3.4, *p* = 0.003). - Regular drinking of tap water was a protective factor for CD (CD = OR 0.6; 95% CI 0.3 to 1, *p* = 0.05).
Amre *et al.* 2007 [11]	Case-control study	Children and adolescents ≤20 years (*n* = 130), newly diagnosed with CD mean age at diagnosis (±SD) 14.2 ± 2.7 years. Healthy controls matched for age and sex (*n* = 202).	- Higher amounts of vegetables (OR = 0.69; 95% CI 0.33 to 1.44, *p* = 0.03), fruits (OR = 0.49; 95% CI 0.25–0.96, *p* = 0.02), fish (OR = 0.46; 95% CI 0.20 to 1.06, *p* = 0.02) and dietary fiber (OR = 0.12; 95%, CI 0.04 to 0.37, *p* < 0.001) protected from CD. - Consumption of LC ω-3 (OR = 0.44; 95%, CI 0.19 to 1.00, *p* < 0.001) were negatively associated with CD. - A higher ratio of LC ω-3/ω-6 fatty acids (OR = 0.32, 95% CI 0.14 to 0.71, *p* = 0.02) were significantly associated with lower risks for CD.
D’Souza *et al.* 2008 [12]	Case-control study	Children and adolescents ≤20 years (*n* = 149), newly diagnosed with CD mean age at diagnosis (±SD) 13.3 ± 2.6 years. Healthy controls matched for age and sex (*n* = 251).	- Meats, fatty foods and desserts (OR = 4.7; 95% CI 1.6 to 14.2) were positively associated with CD. - Vegetables, fruits, olive oil, fish, grains, and nuts were inversely associated with CD in both genders (girls: OR = 0.3; 95% CI 0.1 to 0.9; boys: OR = 0.2; 95% CI 0.1 to 0.5).
Jakobsen *et al.* 2012 [13]	Case-control study	Children and adolescents with IBD (*n* = 118; CD 59, UC 56, IBD-unclassified 3) aged <15 years. Healthy controls matched for age and sex (*n* = 477).	- High sugar intakes were a risk factor for IBD (IBD OR = 2.5; 95% CI 1.0 to 6.2, CD OR = 2.9; 95% CI 1.0 to 8.5). - Protective factors were daily *vs.* less than daily vegetable consumption (CD OR = 0.3; 95% CI 0.1 to 1.0, UC OR = 0.3; 95% CI 0.1 to 0.8) and whole-meal bread consumption (IBD OR = 0.5; 95% CI 0.3 to 0.9, CD OR = 0.4; 95% CI 0.2 to 0.9).

**Table 2:** Clinical studies on efficacy of exclusive enteral nutrition in pediatric CD. CREN = constant rate enteral nutrition, PF = polymeric formula, ED = elemental diet, PCDAI = Pediatric Crohn Disease Activity Index, PEN = partial enteral nutrition, EEN = exclusive enteral nutrition, IFX = infliximab, anti-TNF = anti-tumor necrosis factor, TGFβ2 = transforming growth factor beta 2.

Clinical Studies on Efficacy of Exclusive Enteral Nutrition				
Author/Year	Study Type	Population	Method	Main Findings
Navarro *et al.* 1982 [17]	Clinical trial	Children with active CD (*n* = 17)	Exclusive constant rate enteral nutrition (CREN) using a combination of elemental diet and continuous alimentation for 2–7 months, subsequently CREN used to supplement oral alimentation from 12 to 22 months.	After 7 months of exclusive CREN: all children’s symptoms improved; 100% of children presented moderate disease (Lloyd Still and Green scoring >50).
Fell *et al.* 2000 [23]	Clinical trial	Children with active CD (*n* = 29)	EEN with TGFβ2 enriched PF for 8 weeks.	- After 8 weeks 79% (23/29) of children were in clinical remission. - PCDAI declined with treatment. Median PCDAI at baseline 30 (range 12.5–72.5) declined with treatment by a median of 15 at 2 weeks and 25 at 8 weeks (*p* < 0.00001). - Macroscopic and histological healing in the terminal ileum and colon was associated with a decline in ileal and colonic interleukin-1β. (pre-treatment to post-treatment ratio 0.008 and 0.06: *p* = 0.001, *p* = 0.006).
Afzal *et al.* 2004 [24]	Clinical trial	Children and adolescents with active CD (*n* = 26), mean age 14 years	EEN with PF for 8 weeks.	88.6% achieved clinical remission.
Bannerjee *et al.* 2004 [25]	Clinical trial	Children with active CD (*n* = 12)	EEN with PF for 6 weeks.	Significant improvements in inflammatory markers by day 3 (*p* < 0.05) and in clinical activity index PCDAI by day 7.
Gavin *et al.* 2005 [26]	Retrospective cohort study	Children and adolescents with new onset CD (*n* = 40), aged 6–16 years	EEN with PF for 8 weeks.	All patients improved symptomatically and gained weight after 8 weeks of EEN.
Afzal *et al.* 2005 [27]	Prospective cohort study	Children and adolescents with active CD (*n* = 65), aged 8–17 years. Disease localization: ileal (*n* = 12), ileocolonic (*n* = 39), colonic (*n* = 14).	EEN with PF for 8 weeks.	77% remission rate. Remission rates: Colonic group: 50% (7/14), ileocolon group 82.1% (32/39), ileum group 91.7% (11/12), (χ2 test, *p* = 0.021)). The colonic disease group showed the least fall in PCDAI scores at completion of treatment with EEN (*p* = 0.03), with the lowest remission rate (50%).
Knight *et al.* 2005 [28]	Retrospective cohort study	Children with CD (*n* = 44)	Treatment with EEN as primary treatment for 6–8 weeks.	90% (40/44) of patients responded to EEN with a median time to remission of 6 weeks. Crohn’s disease activity index (CDAI) decreased from pre-EEN to post-EEN, mean values of CDAI not available.
Day *et al.* 2006 [29]	Retrospective cohort study	Children with newly diagnosed CD (group 1, *n* = 15) and with active known long-standing CD (group 2, *n* = 12), mean age 11.8 years	- Group 1: EEN with PF for 6–8 weeks as sole initial therapy - Group 2: EEN with PF for 6–8 weeks in addition to any current medical therapy.	Twenty-four (89%) of 27 children completed their prescribed course of EEN. Nineteen (79%) of 24 children entered clinical remission (80% (12/15) in group 1 and 58% (7/12) in group 2). There was no clear relationship between disease location and response to treatment: 75% (3/4) with isolated small bowel, 72.5% (10/14) with ileocolonic and 67% (6/9) with pancolic disease attained remission (*p* > 0.05). In group 1 successful response to EEN was associated with positive weight gains (average weight gain 4.7 ± 3.5 kg) with mean PCDAI decreasing from 37.1 ± 10.8 to 6.7 ± 5.1 after 8 weeks (*p* < 0.0001). Also in group 2, despite a minor rate of remission, the overall average PCDAI scores significantly fell at 8 weeks (*p* < 0.0001) with an improvement of body weight and in at least one markers of inflammation.
De Bie *et al.* 2013 [30]	Retrospective cohort study	Children with newly diagnosed CD (*n* = 77), median age 13.9 years	Patients received EEN (as either hyperosmolar sip feeds or PF by nasogastric tube) for 6 weeks as remission induction therapy, combined with azathioprine maintenance treatment in 92%.	In patients completing a 6-week course of EEN (58) complete remission was achieved in 71% of patients, partial remission in 26%, and no response in 3%. Complete remission rates were higher in children presenting with isolated ileal/ileocaecal disease and malnutrition.
Grover *et al.* 2016 [31]	Clinical trial	Children with newly diagnosed predominantly luminal CD (*n* = 54), median age 12.4 years	EEN for 6–8 weeks in association with early thiopurine treatment (<3 months from diagnosis). Median duration between pre and post EEN assessments was 60.5 days (IQR 56–69.5)	Post EEN: remission rate (PCDAI < 10) 83% (45/54), biochemical remission (CR*P* < 5 mg/dL) 72% (39/54), complete mucosal healing 33% (18/54). Sustained remission was superior in those with complete mucosal healing *vs.* endoscopic disease 72% (13/18) *vs.* 28% (10/36), *p* = 0.003 at 1 year, 50% (8/16) *vs.* 8% (3/24), *p* = 0.008 at 2 years and 50% (8/16) *vs.* 6% (1/19), *p* = 0.005 at 3 years.

**Table 3:** Clinical studies comparing efficacy between two enteral nutrition regimens. PCDAI = Pediatric Crohn’s Disease Activity Index, PF = polymeric formula, ED = elemental diet, PEN = partial enteral nutrition, EEN = exclusive enteral nutrition, IFX = infliximab, anti-TNF = anti-tumor necrosis factor, TGFβ2 = transforming growth factor beta 2, ARC = absolute risk change.

Clinical Studies Comparing Efficacy between Two Enteral Nutrition Regimens				
Author/Year	Study Type	Population	Method	Main Findings
Akobeng *et al.* 2000 [32]	Randomized controlled trial	Children with active CD (*n* = 18).	Standard PF with a low glutamine content (4% of amino-acid composition, group S) *vs.* glutamine enriched PF (42% of amino acid composition, group G) for 4 weeks.	- No difference in remission rates at week 4 between the two groups’ remission 5/9 (55.5%) in group S, 4/9 (44.4%) in group G (*p* = 0.5). ARC −0.11 (exact 95% CI: −0.57 to 0.35). - Improvement in mean PCDAI was significantly more in group S (*p* = 0.002).
Ludvigsson *et al.* 2004 [33]	Randomized controlled trial	Children with active CD (*n* = 33) involving small bowel, colon and perianal region.	Exclusive EEN with ED (*n* = 16) *vs.* PF (*n* = 17) for 6 weeks.	- Similar remission rates at 6 weeks (ED 11/16 (69%), PF 14/17 (82%); *p* = 0.438). Patients on PF gained more weight compared to ED (*p* = 0.004). ARC 0.14 (exact 95% CI: −0.15 to 0.42).
Johnson *et al.* 2006 [34]	Randomized controlled trial	Children with active CD (*n* = 50) involving small bowel and/or colon.	Patients randomly assigned to receive: - 50% total energy requirements with ED (PEN, *n* = 26) for 6 weeks. - 100% energy requirements with ED (EEN, *n* = 24) for 6 weeks.	Remission rate with PEN was lower than with EEN (PEN 4/26 (15%), EEN 10/24 (42%) *p* = 0.035). Although PCDAI fell in both groups, the reduction was greater with EEN (PCDAI reduction PEN −13, 95% CI (−7 to −19) *p* = 0.001; EEN −26 95% CI (−19 to −33), *p* = 0.001) (*p* = 0.005). ARC = −0.26 (exact 95% CI −0.50 to −0.02).
Rodrigues *et al.* 2007 [35]	Retrospective cohort study	Children with active CD (*n* = 98) involving small bowel and/or colon.	Children received EEN at the time of first presentation either PF (*n* = 45, median age 12.2 years) or ED (*n* = 53, median age 11.8 years).	Remission rates were similar between children receiving PF and ED (ED 64%, 95% CI 51–77 *vs.* PF 51%, 95% CI 37–66, *p* = 0.19). ARC = −0.13 (exact 95% CI −0.32 to 0.06). The use of PF did not affect adherence to EEN but was significantly associated with reduced need for nasogastric tube administration.
Hartman *et al.* 2008 [36]	Retrospective cohort study	Children with CD (*n* = 64) involving small bowel, colon and upper GI tract.	Group 1 (*n* = 28, median age 14 years) and group 2 (*n* = 18, median age 12.7 years) received TGFβ2-enriched PF *vs.* standard PF, respectively, as a supplement to their regular nutrition (35%–50% of total caloric intake), for a median follow-up of 5.3 months for group 1 and 4.5 months for group 2. Group 3 (*n* = 18, median age 12.8 years) without formula supplementation, for a median follow-up of 5.5 months.	Supplementation of the diet with PF (both TGFβ enriched and standard) was associated with a decrease in PCDAI (in group 1 from 34.3 to 15.7, *p* < 0.0001; in group 2 from 35 to 22, *p* = 0.02). No significant decrease in PCDAI was recorded in group 3. Remission rates at follow-up: 57% (16/28, *p* = 0.001) in TGFβ2-enriched PF group, 22.2% (4/18, *p* = 0.03) in standard PF group. Remission rate in group 3 was 22.2 (4/18, *p* = 0.03). ARC = 0.35 (exact 95% CI 0.08 to 0.61). Significant improvements in body mass index (*p* = 0.01) and erythrocyte sedimentation rate (*p* = 0.03) were recorded at follow-up (median 3.4 months) only in the TGFβ2-enriched PF group.
Rubio *et al.* 2011 [37]	Retrospective cohort study	Children with newly diagnosed CD or with a first relapse of an established disease on stable medical treatment (*n* = 106).	Children received EEN with PF for 8 weeks as remission induction therapy either per os (group 1, *n* = 45, mean age 11.3 years) or by continuous enteral route via a nasogastric tube (group 2, *n* = 61, mean age 10.9 years).	Fractionated oral nutritional therapy (group 1) didn’t significantly differ from continuous enteral administration (group 2) in inducing remission (75% *vs.* 85%, respectively, *p* = 0.157). All patients showed a significant decrease in disease severity assessed by PCDAI (*p* < 0.0001) and significant improvements in anthropometric measures and inflammatory indices.
Grogan *et al.* 2012 [38]	Double-blind randomized controlled trial	Children with newly diagnosed CD (*n* = 34).	Children were randomized to ED (*n* = 15, mean age 12.6 years) or PF (*n* = 19, mean age 11.7 years) for 6 weeks and were followed up for 2 years.	No significant difference was recorded between ED and PF in inducing remission (93% 14/15 *vs.* 79% 15/19, respectively). ARC = 0.14 (exact 95% CI −0.08 to 0.37). One-third of children maintained remission at 2 years.
Lee *et al.* 2015 [39]	Prospective study	Children with active CD (*n* = 90).	Children were treated with anti-TNF (*n* = 52), with EEN (*n* = 22), and with PEN plus ad lib diet (*n* = 16) for 8 weeks.	Clinical remission (final PCDAI ≤ 10) was achieved by 50% on PEN, 76% EEN, and 73% anti-TNF (*p* = 0.08). ARC= −0.15 (exact 95% CI −0.47 to −0.16). Mucosal healing (estimated by fecal calprotectin ≤ 250 μg/g) was achieved with PEN in 14%, EEN 45%, and anti-TNF 62% (*p* = 0.001). ARC = −0.25 (exact 95% CI −0.52 to 0.02).

**Table 4:** Clinical studies comparing exclusive enteral nutrition to corticosteroids. PCDAI = Pediatric Crohn’s Disease Activity Index, ED = elemental diet, PEN = partial enteral nutrition, CS = corticosteroids, EEN = exclusive enteral nutrition, IFX = infliximab, anti-TNF = anti-tumor necrosis factor, TGFβ2 = transforming growth factor beta 2, ARC = absolute risk change.

Clinical Studies Comparing Exclusive Enteral Nutrition to Corticosteroids				
Author/Year	Study Type	Population	Method	Main Findings
Sanderson *et al.* 1987 [18]	Randomized controlled trial	Children and adolescents with active CD aged 8.6–17.2 years (*n* = 17) involving the small bowel.	- 8 children treated with CS - 9 children treated with exclusive ED via nasogastric tube	- Disease activity (Lloyd–Still activity index) of the children improved significantly in both ED and PF groups after 6 weeks (*p* < 0.01). Growth velocity improved more in the ED group
Thomas *et al.* 1993 [40]	Randomized controlled trial	Children with active CD (*n* = 24): - 8% (2/24) confined to the small bowel - 29% (7/24) had ileal ± caecal involvement - 25% (6/24) ileocolic disease - 38% (9/24) disease confined to the colon	Children randomized to receive ED (*n* = 12) or CS (*n* = 12) for 4 weeks.	- Similar improvement in disease activity (PCDAI) and remission duration in both groups regardless of site of disease. In CS group activity index at baseline: 74, at week 4: 85, median change +11, (*p* < 0.01); in ED group activity index at baseline: 77, at week 4: 88, median change +11 (*p* < 0.01). - Growth velocity significantly better in ED group compared to CS group.
Ruuska *et al.* 1994 [41]	Randomized controlled trial	Children with new onset or relapsing CD (*n* = 19). Ten children had widespread disease affecting both colon and small intestine; in three the disease was limited to the colon and rectum, and six children had only small bowel disease.	- 10 children treated with a whole-protein based formula through a nasogastric tube for 11 weeks -9 children received high dose CS for 11 weeks	- Similar improvements of PCDAI index, clinical symptoms and inflammatory markers within 2 weeks of treatment in both groups. After the end of the follow-up period 2 months after cessation of the treatment, PCDAI was still low in both groups (PCDAI 11.9 ± 7.9 in enteral diet group and 14.3 ± 9.6 in CS group) - During the routine follow-up after the trial (0.3–2.5 years, mean 1.3 years), five of the CS group 55.5% (5/9) whereas only one from the enteral group 10% (1/10) experienced a clinical relapse. ARC = −0.46 (exact 95% CI −0.8 to −0.08).
Terrin *et al.* 2002 [42]	Randomized controlled trial	Children with active CD (*n* = 20), aged 7–17 years, involving the terminal ileum and different areas of the colon; no fistulae or strictures were detected.	- Group A: CS and mesalazine (*n* = 10) - Group B: enteral nutrition group treated with extensively hydrolyzed formula for 8 weeks (*n* = 10)	- Clinical remission was achieved in 90% (9/10) of patients in group B but only in 50% (5/10) in corticosteroid group (*p* < 0.01). ARC = 0.40 (exact 95% CI 0.04 to 0.76). Both treatments were effective in reducing PCDAI scores (baseline group A 32.0 ± 4.7, group B 34.0 ± 4.3; at week 8 group A 13.0 ± 5.18, group B 7.2 ± 3.15, *p* < 0.01), endoscopic scores (baseline group A 3.7 ± 0.48, group B 3.8 ± 0.42; at week 8 group A 2.4 ± 0.96, group B 1.1 ± 0.87, *p* < 0.01) and histological scores (baseline group A 3.3 ± 0.67, group B 3.5 ± 0.52; at week 8 group A 2.5 ± 0.52, group B 1.3 ± 0.82, *p* < 0.05 for group A, *p* < 0.01 for group B). Group B had significantly lower post-trial PCDAI scores than the CS group (PCDAI scores change group B 14.6 ± 3.6, *p* < 0.01, group A 24.8 ± 4.4, not significant))
Borrelli *et al.* 2006 [43]	Randomized controlled trial	Children with active naïve CD (*n* = 37).	- 19 children received EEN with PF for 10 weeks - 18 children received oral CS for 10 weeks	At week 10 the remission rate was comparable between two groups: 15/19 (79%, 95% CI 56–92) in PF group and 12/18 (67%, 95% CI 44–84) in CS group (*p* = 0.4, not significant). ARC = 0.12 (exact 95% CI −0.16 to 0.40). The proportion of children showing mucosal healing was significantly higher in the PF (14/19, 74%; 95% CI 51 to 89) than the CS group (6/18, 33%; 95% CI 16 to 57; *p* < 0.05). ARC = 0.40, (exact 95% CI 0.11 to 0.70). At week 10 both endoscopic and histologic scores significantly decreased only in PF group. For endoscopic score: in PF group pre-trial 12.9 ± 0.8, post-trial 5.9 ± 0.5, *p* < 0.001, in CS group pre-trial 12.9 ± 0.9, post-trial 9.8 ± 1.3, not significant. For histologic scores: in PF group ileum score pre-trial 10.4 ± 0.4, post-trial 3.8 ± 0.5, *p* < 0.001, in CS group pre-trial 11.0 ± 04, post-trial 9.6 ± 0.7, not significant.
Berni Canani *et al.* 2006 [44]	Retrospective cohort study	Children with newly diagnosed CD (*n* = 47), mean age 12.1 years.	Children received nutritional therapy (NT) for 8 weeks as - Polymeric formula (*n* = 12) - Semi-elemental diet (*n* = 13) - Elemental diet (*n* = 12) Ten subjects received oral CS for 8 weeks.	Similar clinical remission rates were observed after 8 weeks of treatment: 86.5% (32/37) receiving NT *vs.* 90% (9/10) treated with CS. ARC = −0.04 (exact 95% CI −0.25 to 0.18). Improvement in mucosal inflammation occurred in 64.8% (26/37) of patients on NT and 40% (4/10) of children on CS (*p* < 0.05).
Soo *et al.* 2013 [45]	Retrospective cohort study	Children with newly diagnosed CD (*n* = 105).	Children received either EEN (*n* = 36, mean age 12.9 years) or corticosteroids (*n* = 69, mean age 11.2 years) as induce remission therapy	Remission rate similar in two groups 88.9% (32/36) in the EEN group *vs.* 91.3% (63/69) in the CS group (*p* = 0.73) at 3 months). ARC= −0.02 (exact 95% CI −0.15 to 0.10). Relapse rate (40.6% *vs.* 28.6%), similar in both treatment groups (*p* = 0.12) over 12 months).
Luo *et al.* 2015 [46]	Retrospective cohort study	Children with newly diagnosed mild to moderate CD.	Children received either EEN (*n* = 10; median age 11.6 years) or CS (*n* = 18; median age 11.1 years) for 8 weeks.	The remission rate in EEN group was significantly higher than that in CS group (90.0% *vs.* 50.0%, respectively, *p* < 0.05).
Grover *et al.* 2015 [47]	Retrospective analysis of records	Children with newly diagnosed CD (*n* = 89) involving ileal, ileocolonic, and colonic sites.	Children received either EEN (*n* = 43; median age 13 years) or CS (*n* = 46; median age 11.5 years) as remission induction therapy together with an early use of thiopurines (within 6 months from diagnosis) as maintenance therapy. They were followed up for at least 2 years.	Choice of EEN over CS induction was associated with reduced linear growth failure (7% *vs.* 26%, *p* = 0.02), CS dependency (7% *vs.* 43%, *p* = 0.002), and improved primary sustained response to IFX (86% *vs.* 68%, *p* = 0.02).

**Table 5:** Novel nutritional approaches for induction of remission in pediatric IBD. SCD = Specific Carbohydrate Diet. CD = Crohn’s Disease, PEN = partial enteral nutrition, PCDAI = Pediatric Crohn’s Disease Activity Index, IR = incidence rate.

Novel Nutritional Approaches for Induction of Remission in Pediatric IBD				
Author/Year	Study Type	Population	Method	Main Findings
Gupta *et al.* 2013 [48]	Retrospective cohort study	Children with active CD (*n* = 23), mean age 12.8 years.	Enteral nutrition providing 80%–90% of caloric needs, remaining calories from normal diet	Induction of remission achieved in 65% of cases and response in 87% of cases at a mean follow-up of 2 months.
Suskind *et al.* 2014 [49]	Retrospective cohort study	Children and adolescents with active CD (*n* = 10), age range 7–16 years	SCD as treatment of active CD (either soon after diagnosis, or as second line therapy if steroid dependent or failure of mesalazine treatment). Duration of dietary therapy: 5–30 months.	Symptoms of all patients resolved at a routine clinic visit 3 months after initiating the diet. Laboratory indices and fecal calprotectin either normalized or significantly improved at the follow-up clinic visits.
Cohen *et al.* 2014 [50]	Clinical trial	Children and adolescents with active CD, mean age 13.6 years (*n* = 9).	SCD for 101% caloric needs, for 12 and 52 weeks.	At both: 12 week and 52-week endpoint s, there was clinical improvement assessed by PCDAI. In 6/10 patients (60%) remission was achieved by week 12. IR = 0.60 (exact 95% CI 0.26 to 0.88). Mucosal healing (Lewis score < 135) was observed in 40% (4/10) of patients at week 12. IR = 0.40 (exact 95% CI 0.12 to 0.74). 80% showed significant mucosal improvement at week 12 when compared to baseline (*p* = 0.012). IR = 0.80 (exact 95% CI 0.44 to 0.97).
Sigall-Boneh *et al.* 2014 [51]	Clinical trial	Children and young adults with active CD *n* = 47 (mean age 16.1 ± 5.6 years, children *n* = 34)	PEN with a CD exclusion diet + 50% polymeric formula for 6 weeks.	Response and remission was obtained in 37 (78.7%) and 33 (70.2%) patients respectively. IR = 0.79 (exact 95% CI 0.64 to 0.89). Remission was obtained in 70% of children and 69% of adults. IR = 0.70 (exact 95% CI 0.55 to 0.83).

**Table 6:** Nutrition in maintenance of disease remission. ED = elemental diet, MEN = maintenance enteral nutrition, EEN = exclusive enteral nutrition, SCD = specific carbohydrate diet, CR*P* = C-reactive protein, BMI = body mass index, 5-ASA = 5-aminosalycilates, ARC = risk change.

Nutrition in Maintenance of Disease Remission in Pediatric IBD				
Author/Year	Study Type	Population	Method	Main Findings
**PEN**				
Belli *et al.* 1988 [54]	Clinical trial	Children and adolescents with CD (*n* = 8) aged 9.8–14.2 years	- 8 children treated with chronic Intermittent ED for 1 month out of 4, over the course of 1 year - 4 children treated with conventional medical treatment	- CD activity index and prednisone intake decreased significantly in patients receiving ED therapy when compared with controls on conventional medical therapy (*p* < 0.05).
Duncan *et al.* 2014 [55]	Clinical trial	Children and adolescents newly diagnosed CD (*n* = 59) aged 2.5–16.33 years	Patients newly diagnosed CD who commenced EEN for 8 weeks, than followed up: - 11/59 poor response to EEN, switched to steroids. 48/59 completed 8 weeks with EEN and achieved remission - 15/48 continued MEN, post EEN completion. Duration of MEN ranged 4–14 months (mean 10.8 months)	- Remission rates at 1 year in patients continuing MEN were 60% (9/15), compared to 15% (2/13) in patients taking no treatment (*p* = 0.001) and 65% (13/20) in patients taking azathioprine (*p* = 0.14).
Wilschanski *et al.* 1996 [61]	Retrospective cohort study	Children and adolescents (*n* = 65) aged 7–17 years	After induction of remission of CD with EEN: - Group 1: patients (*n* = 28) continued nocturnal nasogastric supplementary feeding combined with a normal diet - Group 2: patients (*n* = 19) stopped nocturnal supplements after the remission	- Higher relapse rate in group 2 *vs.* group 2 at 6 and 12 months (At 6 months relapse rate of group 2: 78% (15/19) *vs.* 17.8% (5/28) of group 1, *p* < 0.02; at 12 months relapse rate of group 2 78.9% (15/19) *vs.* group 1 42.8% (12/28), *p* < 0.02). - Mean changes in height velocity was greater for group 1 (2.87 cm/year) compared to group 2 (0.4 cm/year), p =0.057.
Obih *et al.* 2015 [63]	Retrospective cohort study	Children affected by IBD (*n* = 26), CD (*n* = 20) or UC (*n* = 6) aged 1.5–19 years	- Group of patients (*n* = 26) who followed a SCD for more than 2 weeks (mean duration 9.6 ± 10.1 months). 15/26 patients were on concurrent medication with SCD; 11/26 were not on IBD-related drugs - Children with IBD (*n* = 10), CD (*n* = 7), UC (*n* = 3) who did not follow SCD but only standard medical therapy	- In SCD group PCDAI improved from 32.8 ± 13.2 at baseline to 20.8 ± 16.6 by week 4 ± 2 w and 8.8 ± 8.5 by month 6. - The mean Pediatric Ulcerative Colitis Index (PUCAI) decreased from baseline 28.3 ± 10.3 to 20.0 + 17.3 at week 4 ± 2 w and to 18.3 ± 31.7 at month 6. - Significant improvement of Crohn’s disease activity index, CRP, and calprotectin in SCD group respect to control group (*p* = 0.03, 0.03, and 0.03, respectively) - lower BMI, height and weight in SCD group than in control (*p* = 0.01, 0.03, and 0.009, respectively)
**Curcumin**				
Hanai H *et al.* 2006 [69]	Randomized double-blind, placebo-controlled trial	Adolescents and adults with quiescent UC aged 13–65 years (*n* = 89)	- 43 patients received curcumin 2 g/day plus sulfasalazine or mesalazine; - 39 patients received placebo plus sulfasalazine or mesalazine	- Relapse rate at 6 months of therapy was lower for curcumin group compared to placebo group: - 2/43 (4.65%) in curcumin group *vs.* 8/39 (20.51%) in placebo group, *p* = 0.049. ARC = −0.16 (exact 95% CI −0.30 to −0.02). - Curcumin improved both clinical activity index (CAI, *p* = 0.038) and endoscopic index (EI, *p* = 0.0001). - High tolerability and no serious side effect associated to curcumin
Suskind *et al.* 2013 [71]	Clinical trial	Children and adolescents (*n* = 11) aged 11–18 years affective by CD (*n* = 6) or UC (*n* = 5).	All patients, in addition to their standard IBD therapy, received increasing doses of curcumin, up to 2 g twice daily for 3 weeks	- High tolerability of curcumin without side effects except for increase in gassiness which was consistently reported in two patients. Three patients had lowering of PUCAI/PCDAI scores. - No patients had IBD relapse or worsening of symptoms.
***n*-3 PUFAs**				
Romano *et al.* 2005 [82]	Double-blind, randomized, placebo-controlled study	Children and adolescents affected by CD (*n* = 38) in remission, aged 5–16 years and treated for 12 months.	- Group 1 (*n* = 18): patients treated with 5-ASA (50 mg/kg/day) and omega-3 fatty acids for 12 months. - Group 2 (*n* = 20): patients treated with 5-ASA (50 mg/kg/day) plus placebo (olive oil in capsules) for 12 months.	Number of patients who relapsed at 12 months was significantly lower in Omega-3 fatty acid group than in patients receiving placebo (relapse rate group 1 11/18 (61%), group 2 19/20 (95%); *p* < 0.001). ARC −0.34 (exact 95% CI −0.58 to −0.09).

**Table 7:** Health benefits of nutritional therapy in pediatric IBD. ED = elemental diet, REE = resting energy expenditure, IGF-1 = insulin like growth factor 1, IGFBP-3 = insulin like growth factor binding protein, EEN = exclusive enteral nutrition, corticosteroids = CS.

Health Benefits of Nutritional Therapy in Pediatric IBD				
Author/Year	Study Type	Population	Method	Main Findings
Azcue *et al.* 1997 [83]	Clinical trial	Children and adolescents affected by CD (*n* = 24); malnourished adolescents with anorexia nervosa (*n* = 19); healthy control subjects (*n* = 22)	- Group of patients (*n* = 12) treated with nocturnal enteral nutrition via nasogastric tube for 5–6 weeks, then 1 night a week for 2 months - Group of patients (*n* = 12) treated with prednisolone - control group (*n* = 22), healthy subjects - group of malnourished adolescents with anorexia nervosa (*n* = 19)	- All body compartments and REE increased significantly in enteral nutrition group compared to patients treated with corticosteroids. In enteral nutrition group REE (kcal/day) from 1153 ± 283 at baseline to 1415 ± 535 at 1 month post-treatment; in prednisolone group REE at baseline 1380 ± 308 to 1432 ± 265 at one month post-treatment. For lean body mass (LBM % weight) in enteral nutrition group 86.6 ± 8.9 at baseline to 88.8 + 9.9 at one month post-treatment, in prednisolone group 87.5 ± 9.4 at baseline to 79.1 ± 9.4 at one month post-treatment. - Significant height increase in enteral nutrition group compared with prednisolone group (*p* < 0.01).
Beattie *et al.* 1998 [86]	Clinical trial	Children and adolescents affected by CD (*n* = 23)	- Study A: 14 patients treated with EEN for 8 weeks, then gradual reduction of nutritional support over 2 months - Study B: 9 patients treated with intestinal resection	Study A - Significant weight gain for all patients treated with EEN after 8 and 16 weeks, compared to pre-treatment values (weight SDS at baseline= -0.9 (−1.3 to −0.5), weight SDS at week 8 = −0.5 (−0.9 to 0), at 16 weeks weight SDS = −0.6 (−1.1 to 0) (*p* < 0.05) - Significant increase of median IGF-1 and IGFBP-3 at 2, 8, and 16 weeks in EEN group compared to pre-treatment values (*p* < 0.01) Study B - No significant changes in auxological data, IGF-1 and IGFBP-3 in patients who had surgical resection compared to pre-treatment values
Wilschanski *et al.* 1996 [61]	Retrospective cohort study	Children and adolescents (*n* = 65) aged 7–17 years	After induction of remission of CD with EEN: - Group 1: patients (*n* = 28) continued nocturnal nasogastric supplementary feeding combined with a normal diet - Group 2: patients (*n* = 19) stopped nocturnal supplements after the remission	- Mean changes in height velocity was greater for group 1 (2.87 cm/year) compared to group 2 (0.4 cm/year), *p* = 0.057.
Belli *et al.* 1988 [54]	Clinical trial	Children and adolescents with CD (*n* = 8) aged 9.8–14.2 years	- 8 children treated with chronic intermittent enteral ED for 1 month out of 4, over the course of 1 year - 4 children treated with conventional medical treatment	- Significant height and weight gains in the ED group *vs.* controls. Weight gain for ED group 6.9 ± 1.5 kg; 209.8% ± 41.9%; weight gain for control group −0.9 + 1.6 kg; −9.8% ± 52.6% (*p* < 0.01). Height changes in ED group 7.0 + 0.8 cm; 126.0% ± 11.8% of ideal predicted. Height change in control group 1.7 + 0.8 cm; 28.7% ± 13.1% (*p* < 0.01).
Berni Canani *et al.* 2006 [44]	Retrospective study	Children and adolescents affected by active CD (*n* = 47)	Children received nutritional therapy (NT) for 8 weeks as - Polymeric formula (*n* = 12) - Semi-elemental diet (*n* = 13) - Elemental diet (*n* = 12) Ten subjects received oral CS for 8 weeks.	- Significant improvement of serum albumin and iron levels in NT compared to CS group. In NT group elemental: albumin at baseline 13.14 ± 0.47 to 3.98 ± 0.36 at 8 weeks, *p* < 0.001; NT group semi-elemental: albumin at baseline 3.13 + 0.40 to 3.88 + 0.26 at 8 weeks, *p* < 0.001; NT group polymeric: albumin at baseline 3.09 + 0.39 to 3.86 + 0.38 at 8 weeks, *p* < 0.001)). In CS group: albumin at baseline 3.37 ± 0.24 to 3.40 ± 0.22 after 8 weeks, *p* = 0.28). In NT group elemental: iron at baseline 31.25 ± 20.4 to 72.17 ± 20.4 at 8 weeks, *p* = 0.001; NT group semi-elemental: iron at baseline 29.46 + 23.7 to 66.69 + 19.8 at 8 weeks, *p* = 0.002; NT group polymeric: iron at baseline 32.58 + 24.0 to 69.25 + 29.6 at 8 weeks, *p* = 0.004)). In CS group: iron at baseline 20.80 ± 15.5 to 35.80 ± 16.0 after 8 weeks, *p* = 0.01). Linear growth recovery was superior in nutritional group compared to CS group (*p* < 0.05).
Motil *et al.* 1982 [87]	Clinical trial	Adolescents affected by CD	- 6 patients affected by CD received nutritional supplements for 7 months - 5 healthy control subjects	Increase of linear and ponderal growth velocities in patients treated with nutritional support (Height gain cm/month pre-supplements 0.10 ± 0.08, post-supplements 0.50 + 0.16; weight gain kg/month pre-supplements 0.21 ± 0.09, post-supplements 1.22 ± 0.25). Achievement of weight and height gain similar to control group levels after the 7 months treatment with nutritional supplements (height gain cm/month 0.38 ± 0.12, weight gain kg/month 0.40 + 0.17).
O’Morain *et al.* 1983 [88]	Clinical trial	Children and adolescents affected by CD (*n* = 15) aged 6–20 years	14 patients received ED as the main energy source for 4 weeks; 1 received corticosteroids.	Improvement of nutritional status, weight, and height gain in children receiving ED.
Whitten *et al.* 2010 [94]	Clinical trial	Children newly diagnosed CD (*n* = 23) compared to a healthy control group (*n* = 20)	Children newly diagnosed with CD received 8 weeks EEN for induction of remission	Normalization of serum markers of bone turnover after EEN therapy. CTX levels at diagnosis 2.967 ± 0.881 ng/mL and after EEN 2.260 ± 0.547 ng/mL, *p* = 0.002. BAP levels at diagnosis 51.24 ± 31.31 microg/L and after EEN 64.82 ± 30.51 microg/L, *p* = 0.02.
Polk *et al.* 1992 [89]	Clinical trial	Adolescents affected by CD (*n* = 6)	All patients received enteral nutrition via nasogastric tube during night 1 out of 4 months for 1 year. Then the patients received a 2-week exclusion diet and a 2-week low-residue diet for 2 months, before re-starting a normal diet.	Significant increase of weight, height, IGF-1, and albumin; decrease of steroid use and disease activity compared to pretreatment values
